# Supplementary figures and images for: Reformulating Couscous with Sprouted Buckwheat: Physico-Chemical Properties and Sensory Characteristics Assessed by E-Senses
Source: Foods. 2023 Sep 26;12(19):3578. doi: 10.3390/foods12193578 (PMC10572695; doi:10.3390/foods12193578)

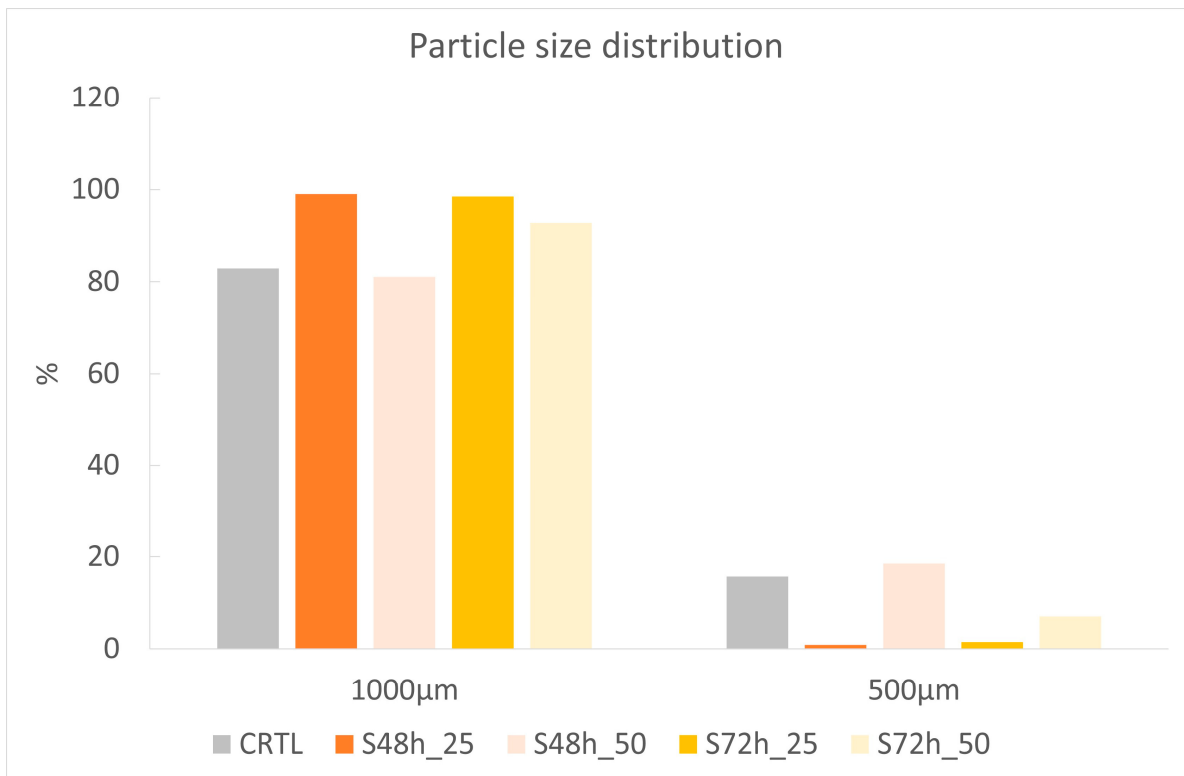

Supplement: Supplementary file 1 [file foods-12-03578-s001.zip › foods-2626279-supplementary.pdf]
